# Working with patients’ treatment expectations – what we can learn from homeopathy

**DOI:** 10.3389/fpsyg.2024.1398865

**Published:** 2024-05-27

**Authors:** Marcel Wilhelm, Christiane Hermann, Winfried Rief, Manfred Schedlowski, Ulrike Bingel, Alexander Winkler

**Affiliations:** ^1^Department of Clinical Psychology, Philipps-University Marburg, Marburg, Germany; ^2^Department of Clinical Psychology, Justus-Liebig-University Giessen, Giessen, Germany; ^3^Institute of Medical Psychology and Behavioral Immunobiology, University Clinic Essen, Essen, Germany; ^4^Department of Clinical Neuroscience, Osher Center for Integrative Medicine, Karolinska Institutet, Stockholm, Sweden; ^5^Department of Neurology, Center for Translational Neuro-and Behavioral Sciences, University Medicine Essen, Essen, Germany; ^6^Translational Pain Research Unit, University Medicine Essen, Essen, Germany

**Keywords:** treatment expectation, homeopathy, placebo, globules, evidence-based medicine

## Abstract

The usual homeopathic remedy, “globules,” does not contain any pharmacologically active ingredient. However, many patients and practitioners report beneficial effects of homeopathic treatment on various health outcomes. Experimental and clinical research of the last two decades analyzing the underlying mechanisms of the placebo effect could explain this phenomenon, with patients’ treatment expectations as the predominant mechanism. Treatment expectations can be optimized through various factors, such as prior information, communication, and treatment context. This narrative review analyses how homeopathy successfully utilizes these factors. Subsequently, it is discussed what evidence-based medicine could learn from homeopathic practice to optimize treatment expectations (e.g., using an empathic, patient-centered communication style, deliberately selecting objects in practice rooms, or using clear treatment rituals and salient contextual stimuli) and thereby treatment effectiveness. Homeopathic remedy does not work beyond the placebo effect but is recommended or prescribed as an active treatment by those who believe in it. Thus, practitioners need to understand the manner in which homeopathy (as an example of inert treatment) works and are advised to reintegrate its underlying effective placebo mechanisms into evidence-based medicine. This promises to increase treatment efficacy, tolerability, satisfaction, and compliance with evidence-based treatments, and addresses the desires patients are trying to satisfy in homeopathy in an ethical, fully informed way that is grounded in evidence-based medicine.

## Introduction

Homeopathy is widely used and accepted in the general population (Relton et al., 2017; Faisal-Cury and Rodrigues, 2022), frequently supported by impressive case stories (Waisse, 2021; Mahesh et al., 2022). It plays a significant role in health care, despite its questionable scientific foundation (Grimes, 2012). Homeopathy is a subcategory of complementary and alternative medicine (CAM) introduced by the physician Samuel Hahnemann in 1796, using substances that, if they were administered undiluted to healthy subjects, would induce symptoms similar to those a patient is experiencing when suffering from a given disease (Swayne, 2000). Counterintuitively, effectiveness is claimed based on the principle “similia similibus curentur” (“like cures like”; e.g., a typical homeopathic remedy for insomnia is based on caffeine, since it keeps a healthy subject awake), and according to Hahnemann’s ‘law of infinitesimals’, diluting and shaking make the homeopathic remedies not less, but more potent. Typically, homeopathic remedies are substances that are highly diluted in alcohol or water. The process of serial dilution combined with shaking is called potentization (Basu et al., 2017). Given that such solutions have nearly non-detectable concentrations of the “active” compound, these proposed mechanisms contradict basic physiological processes (Grimes, 2012). Systematic reviews and meta-analyses consistently show a lack of convincing evidence for the effectiveness of homeopathic remedies, typically in combination with a high risk of bias in the majority of published studies (Kassab et al., 2009; Mathie et al., 2014, 2018; Ostermann et al., 2024). A thorough and independent evaluation by the National Health Medical Research Council of Australia (NHMRC) concluded that there are no health conditions for which there is reliable evidence that homeopathy is effective (National Health Medical Research Council, 2015). In accordance with the NHMRC finding, a systematic review including randomized controlled trials (RCTs) comparing homeopathy and placebo demonstrated that homeopathic remedies are not superior to placebo treatments (Antonelli and Donelli, 2018). Sigurdson et al. (2023) therefore propose to regard homeopathy as a “null field” that still can produce significant effects compared to placebo on meta-analytic level. Erdfelder et al. (2023) show these effects vanish when accounting for selective publication, suggesting a substantial overestimation of homeopathy’s efficacy in published and highly cited studies.

Such critical evaluations are often misunderstood as evidence that homeopathic remedies do not have any effects at all. In clinical drug trials, new drugs are typically considered effective if the symptom improvement in the drug group is superior to the symptom improvement in a placebo group. Regarding homeopathic remedies, there is a huge discrepancy between symptom improvement as perceived by both patients and practitioners in clinical practice and the actual efficacy in placebo-controlled trials, demonstrating that the effect of homeopathic remedies is not superior to, but rather equivalent to the placebo response (Antonelli and Donelli, 2018). While the difference in symptom improvement between the placebo and the homeopathic remedy group is not statistically significant, a significant pre-to post-symptom improvement can occur within both the placebo and homeopathic remedy groups. Hence, the finding that homeopathic remedies are not more effective than placebo does not mean that they do not work at all at an individual level. They just do not work over and above the well-known placebo effect and the natural course of the underlying symptom and disease that typically ceases in many conditions treated with homeopathic remedies, such as respiratory problems, the common cold, injuries, gastrointestinal complaints, bronchitis, burns, allergies, menstrual symptoms, musculoskeletal complaints, mental health, fatigue, stress, pain, and skin problems (Dossett et al., 2016; Achstetter and Teut, 2018; Whitmont, 2019; Uluocak, 2023).

While homeopathy struggles to provide evidence for its effectiveness (National Health Medical Research Council, 2015; Antonelli and Donelli, 2018), the placebo literature grows fast on how to utilize the proven placebo effect in clinical practice (Caliskan et al., 2024).

### Effectiveness of placebo treatments

Placebo responses are observed in the treatment of psoriatic arthritis (Erre et al., 2022), functional gastrointestinal disorders (Enck and Klosterhalfen, 2020), cancer-related fatigue (Roji et al., 2020), cough (Lee et al., 2005, 2019), uncontrolled persistent asthma (Luc et al., 2019), female sexual dysfunction (Weinberger et al., 2018), and in various other physiological systems and medical conditions (Schedlowski et al., 2015). However, their effect sizes seem to vary between systems and conditions. The effects of positive expectation are particularly strong in the fields of pain and depression, where up to 70% of overall treatment effects can be attributed to placebo effects (Moore et al., 2014; Matsingos et al., 2024). Not only do these effects occur in patient-reported outcomes, but can also be shown in physiological parameters, such as heart rate, blood pressure, coronary diameter, gastric motility, lung function, blood sugar levels, or immune function (Meissner, 2014; Park et al., 2016; Kirchhof et al., 2018).

### Mechanisms mediating the placebo effect

The progress in placebo research in the last decades was ground breaking (Schedlowski et al., 2015; Colloca and Barsky, 2020). According to expert consensus, the overall improvement of health outcomes after administration of an inert treatment in placebo-controlled RCTs is defined as placebo response (Evers et al., 2018). The underlying neurobiological mechanisms are increasingly identified. For example, placebos can induce the release of target-specific transmitters such as dopamine in Parkinson’s disease (Lidstone et al., 2010) or endogenous opioids against pain (Eippert et al., 2009). The placebo response accounts for substantial parts of the drug effect in several conditions, such as insomnia (Winkler and Rief, 2015) or hypertension (Wilhelm et al., 2016). Trials in pain (Dolgin, 2010) or mental disorders (Rief et al., 2009b; Weimer et al., 2015) even show that the responses to placebo treatments and active treatments can be quite similar. While the placebo response encompasses also confounding influences such as the natural course of symptoms or statistical phenomena like regression toward the mean (Bland and Altman, 1994; Hengartner, 2020), the placebo effect refers solely to the symptom change attributable to placebo mechanisms, both psychological and neurobiological. Placebo mechanisms are involved in any kind of (active) treatment, and not restricted to inert pills (Benedetti, 2008). The relationship between the placebo effect and regression toward the mean is unclear. Accumulating clinical and experimental evidence indicates that expectation can substantially modulate the efficacy and tolerability of active medical treatments, including pharmacotherapy. Positive treatment expectation has, for instance, been shown to double the analgesic effect of the opioid, remifentanil (Bingel et al., 2011), and to substantially enhance the effect of the acute antimigraine drug, rizatriptane (Kam-Hansen et al., 2014). Therefore, it seems desirable to utilize placebo mechanisms in clinical practice in order to improve health outcomes.

The pivotal role of expectations as one of the most important factors contributing to placebo responses is widely agreed upon in the field of placebo research. Treatment expectations have a strong influence on medical and psychological health outcomes across a wide range of medical treatments (Petrie and Rief, 2019), best illustrated by RCTs involving placebo treatment groups. Since a placebo treatment does not include a biochemically active compound, changes in health outcome within the placebo groups (the placebo response) cannot be explained by specific properties of a drug, but by patients’ expectations regarding the drug treatment they think they received.

**Figure 1:**
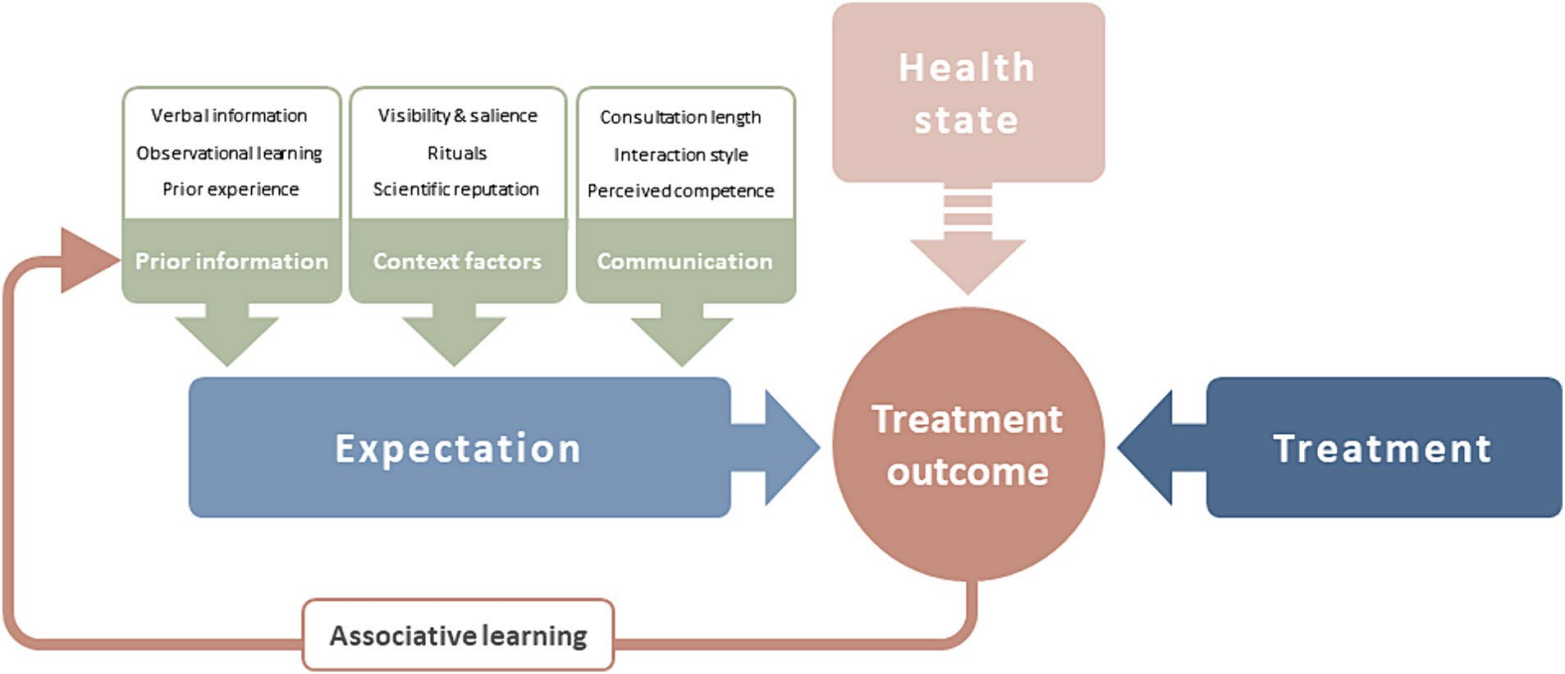
The effect of treatment expectation on treatment outcome [adapted from Bingel (2020)]. Treatment expectation predicts treatment outcome alongside treatment factors and patient’s health state. Expectation is determined by prior information available to the patient, context factors of the treatment situation, and the clinician-patient-communication. Treatment outcome experiences are processed via associative learning to add to prior information.

Recently, Bingel (2020) proposed a model of treatment expectation. According to the model, expectations shape treatment outcomes and are determined by several interacting factors. Treatment expectations themselves are shaped by prior information and context factors—both connected to associative learning—as well as communication (shown in Figure 1). Consequently, Bingel (2020) recommends that systematically targeting treatment expectations should be reintegrated into the biomedical model and current evidence-based treatment regimens. However, while this is a subject of intensive research, it is not yet part of clinical routine, beyond the field of pain, where the systematic use of placebo mechanisms has already been included in treatment guidelines [Deutsche Interdisziplinäre Vereinigung für Schmerztherapie (DIVS), 2008; Klinger, 2010].

Importantly, expectation can also have a negative effect on treatment outcome. Negative expectation, which is linked to the occurrence of unwanted side effects in placebo groups, is being increasingly acknowledged (Colloca and Finniss, 2012; Bingel, 2014, 2020). In fact, the majority of adverse events and symptoms reported by patients in clinical trials may not be caused by the drug itself. This is suggested by the observation that adverse events in placebo arms of drug trials not only resemble those of active treatment in frequency of occurrence, but also in the nature of the symptoms (Rief et al., 2006, 2009a; Amanzio et al., 2009). These effects related to negative treatment expectation are referred to as nocebo responses.

The past century has seen ground breaking advances in the development of new diagnostic tools and increasingly personalized pharmacological (and other) treatments, ranging from the discovery of antibiotics to the use of individually tailored immune treatments for cancer based on the genomic profile of the tumor. These developments have undoubtedly changed the lives of many for the better. Breakthrough achievements in biomedical research now allow us to specifically address underlying pathologies of many diseases and RCTs test whether these new treatments improve health over and above natural fluctuations and other factors unrelated to the intervention. Evidence-based medicine has taken the place of ancient medicine that, due to a lack of the knowledge we have today, largely had to rely on hope and the expectation both of the patient and the healer that an intervention would promote or restore health (Kaptchuk, 2011). Although far from being understood, these forces had an impact on health outcomes and have been considered a pillar of medicine since primeval times. With the advent of modern medicine and its focus on disease-specific pathologies, interest in their influence ceased—ironically, at the peril of those developments that were meant to replace them.

The aim of this narrative review is to use the far-reaching implications of recent insights into the nature of placebo and nocebo effects to elucidate how placebo mechanisms are systematically targeted in homeopathy, often in a much better way than in evidence-based medicine. We postulate a scientifically grounded reintegration of the powerful elements of homeopathy into the biomedical model of health and treatment outcomes in evidence-based medicine and pharmacology to assure that they undergo a scientific evaluation and quality control before implementation in clinical practice in an ethically responsible way.

Taken together, it is of great importance to empower health care practitioners with knowledge on how homeopathy utilizes well-known placebo mechanisms. Practitioners need tools and practices to harness the power of treatment expectation in a way that is grounded in the evidence-based principles of modern medicine. This will optimize treatment efficacy and adherence in large populations and ultimately reduce health care costs while improving patients’ care.

## Key insights

Treatment expectation as the predominant placebo mechanism can be targeted and optimized by various factors. These are interdependent and can be overlapping. We conducted a narrative literature review on experimental placebo research, to (I) summaries the factors that determine treatment expectation and analyses how each of these factors is systematically utilized in homeopathic practice; (II) discuss what modern medicine can learn from homeopathic practice.

### Prior information

Prior information about a treatment has been shown to induce or enhance treatment expectations (Bingel, 2020). Expectations are determined by learning mechanisms (e.g., classical conditioning), verbal information provided by healthcare professionals, and information from other sources like the media (Petrie and Rief, 2019).

Verbal information regarding the expected effects of treatments (e.g., from health care professionals) is omnipresent in health care. Direct verbal communication, as well as written information (e.g., medication leaflets), have been shown to shape patients’ treatment expectation and treatment effectiveness (Camerone et al., 2021; Sondermann et al., 2021). In addition, digital and social media play an important role in providing health-related information (Yetman et al., 2021). This is crucial, as placebo and nocebo responses can be triggered by verbal information provided by non-medical persons such as other patients or people in similar health-related situations (Yetman et al., 2021). To induce a positive treatment expectation it might even be enough to see others receiving an effective treatment (observational learning; Petrie and Rief, 2019).

How to communicate possible medication side effects is a prominent objective of nocebo research (Leibowitz et al., 2021). One promising way is to specifically frame side effects as onset sensations (i.e., symptoms indicating that the treatment is starting to work), which enables practitioners to truthfully inform, while modifying expectations about side effects (Wilhelm et al., 2018; Fernandez et al., 2019; Howe et al., 2019).

#### How does homeopathy use prior information?

Homeopathic practitioners capitalize on direct communication with their patients, often preceded by positive reports from other patients who have recommended the practitioner (Goldman and Cornwell, 2015). Moreover, advertisements describe homeopathic treatment as soft, naturalistic, and holistic (Murdoch et al., 2016). Social media offer additional effective ways to induce positive treatment expectation. Online forums of homeopathic practitioners and patients are characterized by positive examples of successful homeopathic treatments, self-experienced or observed by others. Positive case examples are given by the homeopath (e.g., “it worked before,” “all my patients improved,” or “I use that myself every time I feel sick”) through media articles or social media reports (Narayanaswami et al., 2015). Correspondingly, 58% of CAM patients report that knowing of others who have applied CAM motivated them to use CAM, as well (Thompson et al., 2007). In addition, patients who distrust methods of conventional medicine are more vulnerable to health misinformation and report more positive beliefs about alternative medicine (Scherer et al., 2021). At the same time, patients with lower health literacy seem to have difficulties in correctly predicting the effectiveness of homeopathy and conventional medicine after receiving online information (Wilhelm and Euteneuer, 2021).

Another concept of homeopathy, artificial disease, describes a set of symptoms caused by the administration of a homeopathic remedy during “provings” in healthy volunteers (Ernst, 2016). According to Hahnemann’s theory of “like cures like,” the artificial disease would bring the cure by stimulating the vital forces of the patient to overcome the original disease (Ernst, 2016). From a psychological perspective, homeopathic aggravation (i.e., the temporary deterioration of the patient’s symptoms due to the administration of the optimal remedy) could be interpreted as suggestive onset phenomena. In other words, patients expect a certain worsening of their symptoms at the beginning of their treatment, indicating that the homeopathic remedy successfully stimulated their vital forces to overcome the disease, hence inducing a positive treatment expectation. Studies show that subjective worsening of disease symptoms is as frequent in patients receiving homeopathic remedies as in patients receiving placebos (Grabia and Ernst, 2003). There are also side effects that occur in the context of homeopathic treatment that are presumably nocebo responses (Posadzki et al., 2012). The “like cures like” principle explains these symptoms and therefore helps to support and promote it through circular argument. Another asset of homeopaths’ verbal framing is the notion that if the chosen homeopathic remedy did not work right away, it should be changed (more than once), until the effective remedy (of thousands available) is found (Oberbaum et al., 2003). The so-called repertory, an index of the “homeopathic materia medica,” which contains a collection of descriptions of all symptoms, signs, and emotions experienced by healthy volunteers after ingesting specific homeopathic remedies, is used to identify the optimally matched remedy (“similium”; Teixeira, 2023). This trial-and-error testing enhances patients’ treatment expectations due to believing they have received a well-established remedy for the same “symptom picture” they are experiencing. Hence, homeopathy treatment never fails, and takes advantage of phenomena such as regression toward the mean or spontaneous remission of disease symptoms. For instance, a patient experiencing an exceptionally severe symptom is statistically likely to report symptom improvement, irrespective of intervention, due to regression towards the mean. If coincident with homeopathic treatment, this natural decrease may be erroneously attributed to the efficacy of the remedy. At the same time, this treatment rationale cannot be falsified, as it can explain treatment success as well as a lack of treatment success. This optimizes treatment expectations of the homeopathic practitioner and the patient, while in parallel ignoring a confirmation bias that is inherent in the methodology of homeopathic case histories (Rutten, 2013).

### Context factors

Context factors (e.g., the white coat of the physician, the medical equipment, the medical facility, the shape/size/color of a pill, or the act of swallowing a drug, as well as internal cues like symptoms from the patient’s disease or current mental state), could trigger expectations (Petrie and Rief, 2019). Associative learning can also be involved in developing placebo effects by the association of beneficial drug effects with neutral stimuli (e.g., environmental or gustatory cues), which subsequently evoke the benefit without administration of the active drug (Colloca and Barsky, 2020; Hadamitzky et al., 2020; Aulenkamp et al., 2023). The impact of learning on treatment expectation and treatment outcomes has been investigated in experimental studies showing that positive experiences with treatments in the past amplify placebo responses of subsequent treatments (Colloca, 2019). while prior negative experiences make future treatments less effective (Cadorin et al., 2020).

#### Visibility and salience of the treatment

Pills do not act in a vacuum and the pharmacological effects are influenced by contextual factors, environmental enrichment, and social interactions (Colloca, 2019; Rossettini et al., 2020). This is impressively demonstrated by the so-called open-hidden paradigm. The hidden application of a drug (typically administered by a computer-based device at a predetermined time point without the patient’s knowledge) leads to substantially decreased effectiveness of the drug as compared to the open application of the same drug in an identical dosage when administered by a doctor or a nurse (Colloca et al., 2004). For instance, open application has been shown to double the analgesic effect of the opioid Remifentanil, in comparison to a hidden application (Bingel et al., 2011). Other factors of visibility and salience are the physical characteristics of treatment (e.g., larger pills, stronger taste, higher price), invasiveness (Meissner and Linde, 2018; Jonas et al., 2019), or certain brand names (Buckalew and Coffield, 1982; Abel and Glinert, 2008). Along these lines, it has been shown that a pill, which was provided as an expensive pain killer, was more effective in relieving pain than an inexpensive pill, with both pills being a placebo (Waber, 2008). For instance, invasive interventions (e.g., acupuncture, injections, surgery) induce more pronounced treatment expectations and subsequently greater health benefits than treatments that are less salient and intrusive, such as oral treatments (e.g., pills; Meissner and Linde, 2013; Jonas et al., 2019). Active placebos (i.e., treatments involving the application of a pharmacological substance that induces noticeable (side) effects, but with no effect on the target symptom) can induce stronger placebo effects than inert placebos (Rief and Glombiewski, 2012).

#### Physical environmental factors

Objects such as clothing or décor in health care settings seem to influence health outcomes (Bernstein et al., 2020). Rated quality of care is higher, for example, when the physician attire is more professional, when credentials such as diplomas are displayed on the walls, or when there is a thoughtfully furnished waiting room (Andrade et al., 2013). Hence, a possible modulation of placebo and nocebo effects through objects appears likely. It has already been shown in 1984 that patients undergoing cholecystectomy who were assigned to rooms with a view of nature had shorter postoperative hospital stays, received fewer negative evaluative comments in nurses’ notes, and took fewer potent analgesics than the matched control group of patients in similar rooms with windows facing a brick wall (Ulrich, 1984).

#### Rituals such as the intervention itself

Due to a lack of today’s knowledge, ancient medicine largely relied on the hope and expectation—both of the patient and the healer—that a ritualized intervention would promote or restore health (Kaptchuk, 2011). Although far from being understood, these forces had an impact on health outcomes and have been considered a pillar of medicine since primeval times. Now, as then, a medical treatment consists of several steps to unfold its specific effect. These steps can be described as a treatment ritual (Kaptchuk, 2002). For example, taking a pill, getting an injection, or surgery activates placebo mechanisms (Miller and Colloca, 2010; Benedetti and Amanzio, 2011). The importance of treatment rituals is supported by the observation that diazepam reduces anxiety only in open application, whereas the effect vanishes in hidden application due to no ritual signaling to patients that the treatment has started—thus not activating any treatment expectations (Colloca et al., 2004; Bingel et al., 2011).

##### How does homeopathy use contextual factors?

Homeopaths use objects, such as practice rooms and structures, certificates, or technical instruments. For example, the so-called “vega-test,” which supposedly uses an electrical circuit passing through the patient to measure the body’s electrical energy is used to determine the optimal homeopathic remedy. While scientific tests have shown no validity for the vega-test (Katelaris et al., 1991; Lewith, 2001), the medical instrumentation itself may activate patients’ expectations. In addition, highly specific rituals for taking globules, such as to take them only with a plastic spoon, under no circumstances together with dairy products, or to take 30 globules 12 times a day (Ernst, 2016) might underpin the professionalism of homeopathic treatment and support patients expectations of beneficial treatment. In addition, patients seeking help from homeopaths predominantly start consultations with positive expectations (e.g., homeopathic treatments causing less side effects while supporting the natural healing process; Reid, 2002), enhancing physical and psychological well-being (Guethlin et al., 2010), and also having specific effects on the natural course of the condition (Ernst and Hung, 2011).

In very high potencies (highly diluted), homeopathic remedies can be expensive, and are often paid for by the patients themselves. Well-known homeopaths often charge more for their treatments than regular medical treatments would cost. In their recent cost-effectiveness analysis, Ostermann et al. (2015) concluded that the total treatment costs after 18 months were higher in patients who utilized homeopathic treatment compared to patients who received conventional health care. The common assumption, that homeopathy might help the health care system to save money, ignores that homeopathy typically adds costs since patients do not necessarily reduce their use of regular health care (Ostermann et al., 2015).

### Communication

The quality of the clinician-patient communication in terms of empathy (Kaptchuk et al., 2008), interaction style (Czerniak et al., 2016; Rossettini et al., 2020), and perceived clinical competence of the treatment provider (Howe et al., 2017) increases the placebo effect, therefore improving health outcome. However, even the sheer number of study visits boosts antidepressant placebo effects, as shown in elderly patients with depression (Rutherford et al., 2014) and placebo analgesia in chronic pain patients (Vase et al., 2015). Quality and quantity of clinician-patient communication, in particular communication in an empathic and warm way, prevents nocebo effects (Howe et al., 2017; Zech et al., 2022). However, careful, empathic, and patient-centered communication requires time; yet, the medical care compensation system in many countries forces physicians to see too many patients in too little time. A cross-sectional study in six European countries reported a mean length of consultation in general practice of 10.7 min, varying from 7.6 min in Germany to 15.6 min in Switzerland (Deveugele et al., 2002). Other studies document that physicians in Germany interrupt their patients after an average of 18 s, in the U.S. after 23 s, and that in the U.S. 50% of patients leave the doctor’s office not having understood what the physician has told them (Bodenheimer, 2008).

#### How does homeopathy use communication?

In contrast to the time restrictions in routine medical care, a typical first homeopathic session has a mean consultation length between 60 and 180 min (Grams, 2018). There are also reports of regular sessions of two to three hours in duration (Frank, 2002). Hahnemann explicitly taught his students to let patients finish their reports, no matter how long it takes (Hahnemann and Haehl, 2005). As a consequence, patients often experience more empathy during a consultation with a homeopath in comparison to general practice (Mercer et al., 2002). This results not only from the sheer duration of the consultation but by homeopathy following a collaborative, patient-centered communication approach (Ruusuvuori, 2005; Thompson and Weiss, 2006). The optimal remedy in classical homeopathy is determined based on long and repeated consultations and is individually tailored to each patient. For example, headaches in different patients would be managed using different homeopathic remedies, according to the patient’s individual symptoms, personality, hereditary factors, and medical history. The fact that patients receive a highly individualized remedy instead of a “one drug fits all” treatment for the given symptom is likely to significantly enhance patients’ treatment expectations of a beneficial homeopathic regimen. A homeopath listens patiently to whatever issue might be important to the patient and shows compassion and empathy (Mercer and Reilly, 2004). Patients turning away from conventional medicine and towards homeopathy report that their wish to be in charge of their own health is too often largely ignored by a practitioner writing a prescription and ending the consultation before all problems, concerns, and worries were expressed by the patient (Walach et al., 2005). This competence in building a trustful patient-practitioner relationship and the focus on collaboration are crucial in empowering the patient as a self-reliant partner (Hartog, 2009). Taking into account that quantity and quality of doctor-patient communication is one of the key factors driving the placebo effect, the communication and interaction style of homeopaths seems to be the most effective ingredient in absence of an active substance (Brien et al., 2011).

### The role of deception in placebo treatments and implications for understanding homeopathy

Administering placebos in clinical practice is constrained by ethical and legal concerns regarding the use of deceptive information traditionally associated with a placebo intervention (Miller and Colloca, 2009; American Medical Association, 2012). However, a promising approach to understand placebo effects beyond deception is the use of open-label placebos (OLP), which is considered ethically compatible with the American Medical Association (AMA) principles of medical ethics (American Medical Association, 2008; Miller and Colloca, 2009; Blease et al., 2016). In OLP application, patients are explicitly informed that they receive an inert placebo pill combined with the OLP rationale developed by Kaptchuk et al. (2010). In most OLP studies, this rationale contains information about (1) the effectiveness of placebos, (2) possible mechanisms (i.e., classical conditioning), (3) the role of expectations, and (4) the need to take the pills faithfully, and is provided interactively in the context of an empathic and supportive patient-provider interaction. Several recent RCTs suggest that OLP can effectively improve symptoms in patients suffering from irritable bowel syndrome (Kaptchuk et al., 2010), major depressive disorder (Kelley et al., 2012; Nitzan et al., 2020), allergic rhinitis (Schaefer et al., 2016, 2018), chronic lower back pain (Carvalho et al., 2016; Kleine-Borgmann et al., 2019), cancer-related fatigue (Hoenemeyer et al., 2018; Zhou et al., 2018), and psychological well-being in stressed students (Kleine-Borgmann et al., 2021; Winkler et al., 2023). A recent meta-analysis of 11 OLP studies found a large positive effect size for symptom reduction across different pathologies in OLP groups compared to untreated controls (von Wernsdorff et al., 2021). Furthermore, positive treatment expectancy has been shown to predict OLP effects on several psychological well-being outcomes (El Brihi et al., 2019). Similar to deceptive placebo effects, classical conditioning and expectancy are considered to at least partially account for the OLP effect, but the mechanisms underlying the effectiveness of OLP remain to be elucidated (Charlesworth et al., 2017; Colloca and Howick, 2018; Kaptchuk, 2018; Kaptchuk and Miller, 2018).

Considering that some participants report no or only minor expectancy for improvement before the treatment (Carvalho et al., 2016; Kaptchuk, 2018), unconscious expectations induced by context variables and bodily sensations (e.g., the sensory perception of swallowing a pill) might also contribute to the OLP effect. Furthermore, placebos presumably retrieve classically conditioned pharmacological memories (i.e., the intake of a placebo pill and its sensory features serve as conditioned stimuli that elicit previously acquired conditioned response such as symptom improvements, irrespective of a deceptive or non-deceptive administration; Colloca and Howick, 2018). This assumption is supported by experimental studies investigating conditioned placebo analgesia in standardized heat pain paradigms, in which the conditioned placebo response persisted even after the placebo manipulation was revealed to the participants (Chung et al., 2007; Schafer et al., 2015). Neuroimaging evidence exists for conditioned placebo responses entailing non-conscious conditioned stimuli (Jensen et al., 2012, 2015), and cognitive theories like predictive coding and embodied cognition (Frith, 2011; Shapiro, 2011; Clark, 2016) support the notion of unconscious processes being involved in the OLP effect (Ballou et al., 2017; Kaptchuk, 2018). According to the concept of embodied cognition, the physical interaction with the environment affects cognition and vice versa (Shapiro, 2011, 2014). For instance, the OLP treatment is provided in a professional “medical” context, therefore the same sensory and motor information is impinging on the brain as during an active medical treatment associated with symptom improvement (Ballou et al., 2017; Kaptchuk, 2018). According to predictive coding, even if the brain predicts that a placebo will not lead to symptom improvement, it might adjust the prediction according to the sensory signals (e.g., intake of a capsule in a medical environment), resulting in the release of neurotransmitters in the brain, contributing to symptom improvement (Ballou et al., 2017; Colloca and Howick, 2018).

#### Homeopathy and open-label placebo in comparison

In homeopathy, a treatment rationale is also provided within the context of an empathic and supportive patient-provider relationship. The rationale in homeopathy often contains information about (1) the effectiveness of the homeopathic remedy based on the personal experience of the homeopath, (2) supposed mechanisms (i.e., “like cures like,” “water memory” or “nanoparticles”), (3) the role of expectations (“you have to believe in the self-healing powers of your body to activate the body’s own healing processes”), and (4) the need to take the homeopathic remedy faithfully at the right time (e.g., 15 min before or after tooth brushing) and according to a comprehensive set of rules like using a plastic instead of a metal spoon. Thus, the addressed characteristics of the homeopathic treatment rationale are similar to the OLP rationale. The main difference between the rationales is that the OLP rationale is scientifically grounded, while the homeopathic rationale is not.

The findings on OLP could explain why patients may benefit from homeopathic treatment, even if they do not believe in the effect of the homeopathic remedy. Looking at the findings on embodied cognition, this could even be possible without ever having taken any pharmaceutically active medicine.

#### What can modern medicine learn from homeopathic practice?

Several of the reasons why homeopathy is attractive to homeopathy users, even though treatments are neither scientifically grounded nor evidence-based, might reflect shortcomings of conventional evidence-based medicine. Ernst and Hung (2011) reviewed the literature and extracted six main expectations of patients regarding homeopathy:

- influence on natural history of conditions
- prevention of illness/well-being
- fewer side-effects
- being in control
- symptom relief
- a boost to the immune system

So, how could evidence-based medicine do better at meeting these needs of patients? Most importantly, clinicians should be encouraged (a) to optimize patients’ treatment expectations, (b) to utilize beneficial treatment context effects whenever possible, as well as (c) to adhere to patient-centered communication. Table 1 summarizes recommendations for optimizing treatment effects based on the lessons learned from homeopathy when analyzed within the framework of the placebo effect and its underlying mechanisms as described by Bingel (2020).

**Table 1:** Recommendations for improving evidence-based medicine based on the analysis of how homeopathy works, adapted from Bingel (2014).

**Prior information**
Illustrate that the treatment is individualized for the patients’ specific symptoms, personality, hereditary factors, and history of the disease Actively frame side effects as onset sensations indicating that the treatment is starting to work Use a biopsychosocial (holistic) approach, including the emotional dimensions of an illness
Refer to positive case examples of successful treatments in addition to scientific evidence Use observational learning (e.g., peer-to-peer coaching or video clips with patient responding well to a treatment)
**Contextual factors**
Improve patient-perceived reputation by wearing a lab coat and stethoscope and selecting the objects in your practice rooms (e.g., medical credentials on the wall) Enhance treatment expectation by appropriate use and display of technical instruments Focus the patient’s attention on how the drug will help Use treatment or application rituals (e.g., take only with food, first thing in the morning, etc.) Use salient stimuli and address multiple senses (touching, smell, visual stimuli, noises, etc.) when the treatment is administered Convey that effective treatment is “valuable”
**Communication**
Indicate that you will provide as much time as necessary for the consultation, to fully understand the patient’s problems Adapt an authentic and empathic patient-centered communication style, value patient’s self-reflections about symptoms and possible causes Avoid interrupting the patient’s report Regularly assess and address patients’ anxieties, concerns, and treatment expectations

#### What should not be learned from homeopathy

The true dilemma in homeopathy is that incorrect information (e.g., biologically implausible mechanisms of action, evidence failing to show that homeopathic remedies are more effective than placebo) violate fundamental values of medical ethics like respect for autonomy (capacity to make an informed decision) or non-maleficence in its current practice.

While homeopathy has an image of being entirely free of risk and side effects, there are several areas of concern. First, low potency remedies might be toxic, if the basic tincture is already toxic, such as with arsenic (a popular homeopathic remedy) or strychnine. Therefore, homeopathic remedies should be regulatorily controlled and treated the same way as “allopathic” medicines with respect to quality assurance and pharmacovigilance. Second, if patients use homeopathy for serious clinical conditions instead of conventional therapy or delay treatments for which there is good evidence concerning safety and effectiveness, they put their health at risk. In some extreme cases, homeopaths even assert to treat Ebola, AIDS, and cancer successfully with homeopathic remedies (Fritts et al., 2008; Lindemann, 2015; Johnson et al., 2018). Therefore, a mandatory risk/benefit analysis including empirical evidence for safety and effectiveness is necessary to make a responsible treatment decision.

## Conclusion

This review focused on how placebo mechanisms are utilized in homeopathic practice and how that might offer insights for evidence-based medicine. Importantly, homeopathy is used as an example of how inert treatments can be made effective and how conditions around this treatment should be used. It is crucial to distinguish between the specific modalities of homeopathy, such as the principles of “like cures like” and potentization, which lack empirical support and contradict established scientific understanding, and the nonspecific therapeutic contexts in which homeopathy is practiced. These contexts include prolonged, empathetic patient-clinician interactions and personalized consultations, which have been shown to enhance placebo effects. While the modalities of homeopathy are not scientifically justified and should not be integrated into evidence-based practice, the contextual factors are often overshadowed in conventional medical practice due to various constraints.

Thus, the recommendation is to learn from the manner in which homeopathic care is delivered, but not to integrate homeopathy itself into evidence-based medicine. This approach would ensure that all elements of care desired by patients and recommended by placebo research are included, adhering to rigorous scientific standards and quality control.

In summary, it is the method of care in homeopathy that may offer valuable insights for improving treatment effectiveness in evidence-based medicine through beneficial placebo effects, not the remedies themselves. This perspective is critical for focusing on empirically supported strategies that enhance patient care and treatment outcomes.

## Author contributions

MW: Conceptualization, Methodology, Writing – original draft, Writing – review & editing. CH: Supervision, Writing – review & editing. WR: Supervision, Writing – review & editing. MS: Visualization, Writing – review & editing. UB: Writing – review & editing. AW: Conceptualization, Methodology, Supervision, Validation, Writing – review & editing.

## References

[ref1] Abel G. A. Glinert L. H. (2008). Chemotherapy as language: sound symbolism in cancer medication names. Soc. Sci. Med. 66, 1863–1869. doi: 10.1016/j.socscimed.2007.12.016, PMID: 18243458

[ref2] Achstetter K. Teut M. (2018). Use of self-medication with homeopathy in Germany: results of an online questionnaire survey. Complement. Med. Res. 25, 383–390. doi: 10.1159/000485077, PMID: 30286479

[ref3] Amanzio M. Corazzini L. L. Vase L. Benedetti F. (2009). A systematic review of adverse events in placebo groups of anti-migraine clinical trials. Pain 146, 261–269. doi: 10.1016/j.pain.2009.07.010, PMID: 19781854

[ref4] American Medical Association (2008). Code of Medical Ethics: Opinions on consent, communication & decision making (Chapter 2). Available at: https://www.ama-assn.org/delivering-care/ethics/code-medical-ethics-consent-communication-decision-making

[ref5] American Medical Association (2012). Code of medical ethics of the American Medical Association: Originally adopted at the adjourned meeting of the national medical convention in Philadelphia, may 1847. Chicago, IL: American Medical Association.

[ref6] Andrade C. C. Lima M. L. Pereira C. R. Fornara F. Bonaiuto M. (2013). Inpatients’ and outpatients’ satisfaction: the mediating role of perceived quality of physical and social environment. Health Place 21, 122–132. doi: 10.1016/j.healthplace.2013.01.013, PMID: 23454733

[ref7] Antonelli M. Donelli D. (2018). Reinterpreting homoeopathy in the light of placebo effects to manage patients who seek homoeopathic care: a systematic review. Health Soc. Care Community 27, 824–847. doi: 10.1111/hsc.12681 30456773

[ref8] Aulenkamp J. L. Icenhour A. Elsenbruch S. (2023). Nocebo effects in visceral pain: concept and design of the experimental randomized-controlled pain study 'NoVis'. Front. Psych. 14:1270189. doi: 10.3389/fpsyt.2023.1270189, PMID: 37900300 PMC10603299

[ref9] Ballou S. Kaptchuk T. J. Hirsch W. Nee J. Iturrino J. Hall K. T.. (2017). Open-label versus double-blind placebo treatment in irritable bowel syndrome: study protocol for a randomized controlled trial. Trials 18, 234–215. doi: 10.1186/s13063-017-1964-x, PMID: 28545508 PMC5445390

[ref10] Basu A. Suresh A. K. Kane S. G. Bellare J. R. (2017). A review of machines and devices to potentize homeopathic medicines. Homeopathy J. Fac. Homeopathy 106, 240–249. doi: 10.1016/j.homp.2017.09.002, PMID: 29157473

[ref11] Benedetti F. (2008). Mechanisms of placebo and placebo-related effects across diseases and treatments. Annu. Rev. Pharmacol. Toxicol. 48, 33–60. doi: 10.1146/annurev.pharmtox.48.113006.094711, PMID: 17666008

[ref12] Benedetti F. Amanzio M. (2011). The placebo response: how words and rituals change the patient’s brain. Patient Educ. Couns. 84, 413–419. doi: 10.1016/j.pec.2011.04.034, PMID: 21621366

[ref13] Bernstein M. H. Locher C. Kube T. Buergler S. Stewart-Ferrer S. Blease C. (2020). Putting the ‘art’ into the ‘art of medicine’: the under-explored role of artifacts in placebo studies. Front. Psychol. 11:1354. doi: 10.3389/fpsyg.2020.01354, PMID: 32774310 PMC7387723

[ref14] Bingel U. (2014). Avoiding nocebo effects to optimize treatment outcome. J. Am. Med. Assoc. 312, 693–694. doi: 10.1001/jama.2014.8342, PMID: 25003609

[ref15] Bingel U. (2020). Placebo 2.0: the impact of expectations on analgesic treatment outcome. Pain 161, S48–S56. doi: 10.1097/j.pain.0000000000001981, PMID: 33090739

[ref16] Bingel U. Wanigasekera V. Wiech K. Ni Mhuircheartaigh R. Lee M. C. Ploner M.. (2011). The effect of treatment expectation on drug efficacy: imaging the analgesic benefit of the opioid remifentanil. Sci. Transl. Med. 3:70ra14. doi: 10.1126/scitranslmed.3001244 21325618

[ref17] Bland J. M. Altman D. G. (1994). Statistics notes: some examples of regression towards the mean. BMJ 309:780. doi: 10.1136/bmj.309.6957.780, PMID: 7950567 PMC2541039

[ref18] Blease C. Colloca L. Kaptchuk T. J. (2016). Are open-label placebos ethical? Informed consent and ethical equivocations. Bioethics 30, 407–414. doi: 10.1111/bioe.12245, PMID: 26840547 PMC4893896

[ref19] Bodenheimer T. (2008). Transforming practice. N. Engl. J. Med. 359, 2086–2089. doi: 10.1056/NEJMp0805631 19005190

[ref20] Brien S. Lachance L. Prescott P. McDermott C. Lewith G. (2011). Homeopathy has clinical benefits in rheumatoid arthritis patients that are attributable to the consultation process but not the homeopathic remedy: a randomized controlled clinical trial. Rheumatology 50, 1070–1082. doi: 10.1093/rheumatology/keq234, PMID: 21076131 PMC3093927

[ref21] Buckalew L. W. Coffield K. E. (1982). An investigation of drug expectancy as a function of capsule color and size and preparation form. J. Clin. Psychopharmacol. 2, 245–248. doi: 10.1097/00004714-198208000-00003, PMID: 7119132

[ref22] Cadorin L. Rossettini G. Testa M. Geri T. Palese A. (2020). The awareness of contextual factors, placebo and nocebo effects among nursing students: findings from a cross-sectional study. Nurse Educ. Pract. 42:102670. doi: 10.1016/j.nepr.2019.102670, PMID: 31775083

[ref23] Caliskan E. B. Bingel U. Kunkel A. (2024). Translating knowledge on placebo and nocebo effects into clinical practice. PAIN Rep. 9:e1142. doi: 10.1097/PR9.0000000000001142, PMID: 38533458 PMC10965200

[ref24] Camerone E. M. Piedimonte A. Testa M. Wiech K. Vase L. Zamfira D. A.. (2021). The effect of temporal information on placebo analgesia and nocebo hyperalgesia. Psychosom. Med. 83, 43–50. doi: 10.1097/PSY.0000000000000882, PMID: 33109926

[ref25] Carvalho C. Caetano J. M. Cunha L. Rebouta P. Kaptchuk T. J. Kirsch I. (2016). Open-label placebo treatment in chronic low back pain: a randomized controlled trial. Pain 157, 2766–2772. doi: 10.1097/j.pain.0000000000000700, PMID: 27755279 PMC5113234

[ref26] Charlesworth J. E. G. Petkovic G. Kelley J. M. Hunter M. Onakpoya I. Roberts N.. (2017). Effects of placebos without deception compared with no treatment: a systematic review and meta-analysis. J. Evid.-Based Med. 10, 97–107. doi: 10.1111/jebm.12251, PMID: 28452193

[ref27] Chung S. K. Price D. D. Verne G. N. Robinson M. E. (2007). Revelation of a personal placebo response: its effects on mood, attitudes and future placebo responding. Pain 132, 281–288. doi: 10.1016/j.pain.2007.01.034, PMID: 17368941 PMC2170529

[ref28] Clark A. (2016). Surfing uncertainty: prediction, action, and the embodied mind. Oxford and New York and Auckland: Oxford University Press.

[ref29] Colloca L. (2019). How do placebo effects and patient-clinician relationships influence behaviors and clinical outcomes? PAIN Rep. 4:e758. doi: 10.1097/PR9.0000000000000758, PMID: 31583366 PMC6749893

[ref30] Colloca L. Barsky A. J. (2020). Placebo and nocebo effects. N. Engl. J. Med. 382, 554–561. doi: 10.1056/NEJMra1907805 32023375

[ref31] Colloca L. Finniss D. (2012). Nocebo effects, patient-clinician communication, and therapeutic outcomes. JAMA 307, 567–568. doi: 10.1001/jama.2012.115, PMID: 22318275 PMC6909539

[ref32] Colloca L. Howick J. (2018). Placebos without deception: outcomes, mechanisms, and ethics. Int. Rev. Neurobiol. 138, 219–240. doi: 10.1016/bs.irn.2018.01.005, PMID: 29681327 PMC5918690

[ref33] Colloca L. Lopiano L. Lanotte M. Benedetti F. (2004). Overt versus covert treatment for pain, anxiety, and Parkinson’s disease. Lancet Neurol. 3, 679–684. doi: 10.1016/S1474-4422(04)00908-1, PMID: 15488461

[ref34] Czerniak E. Biegon A. Ziv A. Karnieli-Miller O. Weiser M. Alon U.. (2016). Manipulating the placebo response in experimental pain by altering Doctor’s performance style. Front. Psychol. 7:874. doi: 10.3389/fpsyg.2016.00874, PMID: 27445878 PMC4928147

[ref35] Deutsche Interdisziplinäre Vereinigung für Schmerztherapie (DIVS) (2008). S3-Leitlinie “Behandlung akuter perioperativer und posttraumatischer Schmerzen”. Köln: Dt. Ärzte-Verlag.

[ref36] Deveugele M. Derese A. van den Brink-Muinen A. Bensing J. de Maeseneer J. (2002). Consultation length in general practice: cross sectional study. Br. Med. J. 325, 472–474. doi: 10.1136/bmj.325.7362.472, PMID: 12202329 PMC119444

[ref37] Dolgin E. (2010). Fluctuating baseline pain implicated in failure of clinical trials. Nat. Med. 16:1053. doi: 10.1038/nm1010-1053a, PMID: 20930724

[ref38] Dossett M. L. Davis R. B. Kaptchuk T. J. Yeh G. Y. (2016). Homeopathy use by US adults: results of a National Survey. Am. J. Public Health 106, 743–745. doi: 10.2105/AJPH.2015.303025, PMID: 26890179 PMC4816083

[ref39] Eippert F. Bingel U. Schoell E. D. Yacubian J. Klinger R. Lorenz J.. (2009). Activation of the Opioidergic descending pain control system underlies placebo analgesia. Neuron 63, 533–543. doi: 10.1016/j.neuron.2009.07.014, PMID: 19709634

[ref40] El Brihi J. Horne R. Faasse K. (2019). Prescribing placebos: an experimental examination of the role of dose, expectancies, and adherence in open-label placebo effects. Ann. Behav. Med. Publ. Soc. Behav. Med 53, 16–28. doi: 10.1093/abm/kay011, PMID: 29547962

[ref41] Enck P. Klosterhalfen S. (2020). Placebo responses and placebo effects in functional gastrointestinal disorders. Front. Psych. 11:797. doi: 10.3389/fpsyt.2020.00797, PMID: 33192627 PMC7477083

[ref42] Erdfelder E. Nagel J. Heck D. W. Petras N. (2023). Uncovering null effects in null fields: the case of homeopathy. J. Clin. Epidemiol. 166:111216. doi: 10.1016/j.jclinepi.2023.11.006, PMID: 37996042

[ref43] Ernst E. (2016). Homeopathy-the undiluted facts: Including a comprehensive AZ lexicon. Cham, Switzerland: Springer.

[ref44] Ernst E. Hung S. K. (2011). Great expectations: what do patients using complementary and alternative medicine Hope for? Patient Patient-Centered Outcomes Res. 4, 89–101. doi: 10.2165/11586490-000000000-00000 21766898

[ref45] Erre G. L. Mavridis D. Woodman R. J. Mangoni A. A. (2022). Placebo response in psoriatic arthritis clinical trials: a systematic review and meta-analysis. Rheumatology 61, 1328–1340. doi: 10.1093/rheumatology/keab774, PMID: 34664615

[ref46] Evers A. W. M. Colloca L. Blease C. Annoni M. Atlas L. Y. Benedetti F.. (2018). Implications of placebo and nocebo effects for clinical practice: expert consensus. Psychother. Psychosom. 87, 204–210. doi: 10.1159/000490354 29895014 PMC6191882

[ref47] Faisal-Cury A. Rodrigues D. M. D. O. (2022). Prevalence and associated factors with homeopathy use in Brazil: a population-based study. Cad. Saúde Pública 38:e00261821. doi: 10.1590/0102-311xen261821, PMID: 36169510

[ref48] Fernandez A. Kirsch I. Noël L. Rodondi P. Y. Kaptchuk T. J. Suter M. R.. (2019). A test of positive suggestions about side effects as a way of enhancing the analgesic response to NSAIDs. PLoS One 14:e0209851. doi: 10.1371/journal.pone.0209851, PMID: 30605458 PMC6317829

[ref49] Frank R. (2002). Homeopath & patient–a dyad of harmony? Soc. Sci. Med. 55, 1285–1296. doi: 10.1016/S0277-9536(01)00283-0 12231009

[ref50] Frith C. D. (2011). Making up the mind: How the brain creates our mental world. Malden, Massachusetts: Blackwell.

[ref51] Fritts M. Crawford C. Quibell D. Gupta A. Jonas W. Coulter I.. (2008). Traditional Indian medicine and homeopathy for HIV/AIDS: a review of the literature. AIDS Res. Ther. 5:25. doi: 10.1186/1742-6405-5-25, PMID: 19102742 PMC2637286

[ref52] Goldman A. Cornwell B. (2015). Social network bridging potential and the use of complementary and alternative medicine in later LIFE *. Soc. Sci. Med. 140, 69–80. doi: 10.1016/j.socscimed.2015.07.003, PMID: 26207353 PMC4697732

[ref53] Grabia S. Ernst E. (2003). Homeopathic aggravations: a systematic review of randomised, placebo-controlled clinical trials. Homeopathy 92, 92–98. doi: 10.1016/S1475-4916(03)00007-9, PMID: 12725251

[ref54] Grams N. (2018). Homöopathie neu gedacht. Berlin, Heidelberg: Springer Berlin Heidelberg.

[ref55] Grimes D. R. (2012). Proposed mechanisms for homeopathy are physically impossible: original article. Focus. Altern. Complement. Ther. 17, 149–155. doi: 10.1111/j.2042-7166.2012.01162.x

[ref56] Guethlin C. Walach H. Naumann J. Bartsch H.-H. Rostock M. (2010). Characteristics of cancer patients using homeopathy compared with those in conventional care: a cross-sectional study. Ann. Oncol. 21, 1094–1099. doi: 10.1093/annonc/mdp421, PMID: 19858085

[ref57] Hadamitzky M. Lückemann L. Pacheco-López G. Schedlowski M. (2020). Pavlovian conditioning of immunological and neuroendocrine functions. Physiol. Rev. 100, 357–405. doi: 10.1152/physrev.00033.2018, PMID: 31437089

[ref58] Hahnemann S. Haehl R. (2005). “Organon der Heilkunst: Aude sapere., Neu gesetzte und überarb” in Aufl. d. Ausg. Leipzig 1921 (= 6.Aufl.) ed. R. Haehl (Wiesbaden: Marixverl).

[ref59] Hartog C. S. (2009). Elements of effective communication—rediscoveries from homeopathy. Patient Educ. Couns. 77, 172–178. doi: 10.1016/j.pec.2009.03.021, PMID: 19372024

[ref60] Hengartner M. P. (2020). Is there a genuine placebo effect in acute depression treatments? A reassessment of regression to the mean and spontaneous remission. BMJ Evid. Based Med. 25, 46–48. doi: 10.1136/bmjebm-2019-111161, PMID: 30975717

[ref61] Hoenemeyer T. W. Kaptchuk T. J. Mehta T. S. Fontaine K. R. (2018). Open-label placebo treatment for Cancer-related fatigue: a randomized-controlled clinical trial. Sci. Rep. 8, 2784–2788. doi: 10.1038/s41598-018-20993-y, PMID: 29426869 PMC5807541

[ref62] Howe L. C. Goyer J. P. Crum A. J. (2017). Harnessing the placebo effect: exploring the influence of physician characteristics on placebo response. Health Psychol. 36, 1074–1082. doi: 10.1037/hea0000499 28277699 PMC7608626

[ref63] Howe L. C. Leibowitz K. A. Perry M. A. Bitler J. M. Block W. Kaptchuk T. J.. (2019). Changing patient mindsets about non–life-threatening symptoms during Oral immunotherapy: a randomized clinical trial. J Allergy Clin Immunol Pract 7, 1550–1559. doi: 10.1016/j.jaip.2019.01.022, PMID: 30682576 PMC6511320

[ref64] Jensen K. B. Kaptchuk T. J. Kirsch I. Raicek J. Lindstrom K. M. Berna C.. (2012). Nonconscious activation of placebo and nocebo pain responses. Proc. Natl. Acad. Sci. USA 109, 15959–15964. doi: 10.1073/pnas.1202056109, PMID: 23019380 PMC3465419

[ref65] Jensen K. B. Kirsch I. Odmalm S. Kaptchuk T. J. Ingvar M. (2015). Classical conditioning of analgesic and hyperalgesic pain responses without conscious awareness. Proc. Natl. Acad. Sci. USA 112, 7863–7867. doi: 10.1073/pnas.1504567112, PMID: 25979940 PMC4485119

[ref66] Johnson S. B. Park H. S. Gross C. P. Yu J. B. (2018). Use of alternative medicine for cancer and its impact on survival. J. Natl. Cancer Inst. 110, 121–124. doi: 10.1093/jnci/djx145 28922780

[ref67] Jonas W. B. Crawford C. Colloca L. Kriston L. Linde K. Moseley B.. (2019). Are invasive procedures effective for chronic pain? A systematic review. Pain Med. 20, 1281–1293. doi: 10.1093/pm/pny154, PMID: 30204920 PMC6611529

[ref68] Kam-Hansen S. Jakubowski M. Kelley J. M. Kirsch I. Hoaglin D. C. Kaptchuk T. J.. (2014). Altered placebo and drug labeling changes the outcome of episodic migraine attacks. Sci. Transl. Med. 6:218ra5. doi: 10.1126/scitranslmed.3006175, PMID: 24401940 PMC4005597

[ref69] Kaptchuk T. J. (2002). The placebo effect in alternative medicine: can the performance of a healing ritual have clinical significance? Ann. Intern. Med. 136, 817–825. doi: 10.7326/0003-4819-136-11-200206040-00011, PMID: 12044130

[ref70] Kaptchuk T. J. (2011). Placebo studies and ritual theory: a comparative analysis of Navajo, acupuncture and biomedical healing. Philos. Trans. R. Soc. B Biol. Sci. 366, 1849–1858. doi: 10.1098/rstb.2010.0385, PMID: 21576142 PMC3130398

[ref71] Kaptchuk T. J. (2018). Open-label placebo: reflections on a research agenda. Perspect. Biol. Med. 61, 311–334. doi: 10.1353/pbm.2018.0045, PMID: 30293971

[ref72] Kaptchuk T. J. Friedlander E. Kelley J. M. Sanchez M. N. Kokkotou E. Singer J. P.. (2010). Placebos without deception: a randomized controlled trial in irritable bowel syndrome. PLoS One 5, 1–7. doi: 10.1371/journal.pone.0015591, PMID: 21203519 PMC3008733

[ref73] Kaptchuk T. J. Kelley J. M. Conboy L. A. Davis R. B. Kerr C. E. Jacobson E. E.. (2008). Components of placebo effect: randomised controlled trial in patients with irritable bowel syndrome. Bmj-Br. Med. J. 336, 999–1003. doi: 10.1136/bmj.39524.439618.25, PMID: 18390493 PMC2364862

[ref74] Kaptchuk T. J. Miller F. G. (2018). Open label placebo: can honestly prescribed placebos evoke meaningful therapeutic benefits? BMJ 363:k3889. doi: 10.1136/bmj.k3889, PMID: 30279235 PMC6889847

[ref75] Kassab S. Cummings M. Berkovitz S. van Haselen R. Fisher P. (2009). Homeopathic medicines for adverse effects of cancer treatments. Cochrane Database Syst. Rev. 2016:CD004845. doi: 10.1002/14651858.CD004845.pub2, PMID: 19370613 PMC10422695

[ref76] Katelaris C. H. Weiner J. M. Heddle R. J. Stuckey M. S. Yan K. W. (1991). Vega testing in the diagnosis of allergic conditions. Med. J. Aust. 155, 113–114. doi: 10.5694/j.1326-5377.1991.tb142141.x, PMID: 1857287

[ref77] Kelley J. M. Kaptchuk T. J. Cusin C. Lipkin S. Fava M. (2012). Open-label placebo for major depressive disorder: a pilot randomized controlled trial. Psychother. Psychosom. 81, 312–314. doi: 10.1159/000337053 22854752 PMC3813004

[ref78] Kirchhof J. Petrakova L. Brinkhoff A. Benson S. Schmidt J. Unteroberdörster M.. (2018). Learned immunosuppressive placebo responses in renal transplant patients. Proc. Natl. Acad. Sci. 115, 4223–4227. doi: 10.1073/pnas.1720548115, PMID: 29610294 PMC5910853

[ref79] Kleine-Borgmann J. Schmidt K. Billinger M. Forkmann K. Wiech K. Bingel U. (2021). Effects of open-label placebos on test performance and psychological well-being in healthy medical students: a randomized controlled trial. Sci. Rep. 11, 1–11. doi: 10.1038/s41598-021-81502-2 33483552 PMC7822842

[ref80] Kleine-Borgmann J. Schmidt K. Hellmann A. Bingel U. (2019). Effects of open-label placebo on pain, functional disability, and spine mobility in patients with chronic back pain: a randomized controlled trial. Pain 160, 2891–2897. doi: 10.1097/j.pain.0000000000001683, PMID: 31479068

[ref81] Klinger R. (2010). The potential of the analgetic placebo effect-s3-guideline recommendation on the clinical use for acute and perioperative pain management. Anasthesiologie Intensivmed. Notfallmedizin Schmerzther 45, 22–29. doi: 10.1055/s-0029-1243374, PMID: 20091473

[ref82] Lee P. C. Jawad M. S. Hull J. D. West W. H. Shaw K. Eccles R. (2005). The antitussive effect of placebo treatment on cough associated with acute upper respiratory infection. Psychosom. Med. 67, 314–317. doi: 10.1097/01.psy.0000155667.59662.92, PMID: 15784799

[ref83] Lee S.-E. Lee J.-H. Kim H. J. Lee B.-J. Cho S.-H. Price D.. (2019). Inhaled corticosteroids and placebo treatment effects in adult patients with cough: a systematic review and meta-analysis. Allergy Asthma Immunol. Res. 11:e74. doi: 10.4168/aair.2019.11.6.856 PMC6761077 31552720

[ref84] Leibowitz K. A. Howe L. C. Crum A. J. (2021). Changing mindsets about side effects. BMJ Open 11:e040134. doi: 10.1136/bmjopen-2020-040134, PMID: 33526496 PMC7849892

[ref85] Lewith G. T. (2001). Is electrodermal testing as effective as skin prick tests for diagnosing allergies? A double blind, randomised block design study. BMJ 322, 131–134. doi: 10.1136/bmj.322.7279.131, PMID: 11159567 PMC26588

[ref86] Lidstone S. C. Schulzer M. Dinelle K. Mak E. Sossi V. Ruth T. J.. (2010). Effects of expectation on placebo-induced dopamine release in Parkinson disease. Arch. Gen. Psychiatry 67, 857–865. doi: 10.1001/archgenpsychiatry.2010.88, PMID: 20679593

[ref87] Lindemann O. (2015). Bericht über den Einsatz der LMHI in Liberia im Herbst 2014. Allg. Homöopath. Ztg. 260, 18–21. doi: 10.1055/s-0041-107298

[ref88] Luc F. Prieur E. Whitmore G. Gibson P. G. Vandemheen K. L. Aaron S. D. (2019). Placebo effects in clinical trials evaluating patients with uncontrolled persistent asthma. Ann. Am. Thorac. Soc. 16, 1124–1130. doi: 10.1513/AnnalsATS.201901-071OC, PMID: 31063408

[ref89] Mahesh S. Habchi O. Vithoulkas G. (2022). Cervical intraepithelial neoplasia managed with classical homeopathy: a case report. Integr. Med. Rep. 1, 240–248. doi: 10.1089/imr.2022.0067

[ref90] Mathie R. T. Lloyd S. M. Legg L. A. Clausen J. Moss S. Davidson J. R.. (2014). Randomised placebo-controlled trials of individualised homeopathic treatment: systematic review and meta-analysis. Syst. Rev. 3:142. doi: 10.1186/2046-4053-3-142, PMID: 25480654 PMC4326322

[ref91] Mathie R. Ulbrich-Zürni S. Viksveen P. Roberts E. Baitson E. Legg L.. (2018). Systematic review and Meta-analysis of randomised, other-than-placebo controlled, trials of individualised homeopathic treatment. Homeopathy 107, 229–243. doi: 10.1055/s-0038-1667129, PMID: 30121049

[ref92] Matsingos A. Wilhelm M. Noor L. Yildiz C. Rief W. Hofmann S. G.. (2024). Hype or hope? High placebo response in major depression treatment with ketamine and esketamine: a systematic review and meta-analysis. Front. Psych. 15:1346697. doi: 10.3389/fpsyt.2024.1346697, PMID: 38525254 PMC10957753

[ref93] Meissner K. (2014). Placebo responses on cardiovascular, gastrointestinal, and respiratory organ functions. Placebo, 225, 183–203. doi: 10.1007/978-3-662-44519-8_11, PMID: 25304533

[ref94] Meissner K. Linde K. (2013). “What are the best placebo interventions for the treatment of pain?” in Placebo and Pain: From Bench to BedsideII. eds. L. Colloca, M. A. Flaten, and K. Meissner. Vol. 1. (Academic Press), 235–242.

[ref95] Meissner K. Linde K. (2018). Are blue pills better than green? How treatment features modulate placebo effects. Int. Rev. Neurobiol. 139, 357–378. doi: 10.1016/bs.irn.2018.07.014, PMID: 30146054

[ref96] Mercer S. W. Reilly D. (2004). A qualitative study of patient’s views on the consultation at the Glasgow homoeopathic hospital, an NHS integrative complementary and orthodox medical care unit. Patient Educ. Couns. 53, 13–18. doi: 10.1016/S0738-3991(03)00242-8, PMID: 15062899

[ref97] Mercer S. W. Reilly D. Watt G. C. M. (2002). The importance of empathy in the enablement of patients attending the Glasgow homoeopathic hospital. Br. J. Gen. Pract. 52, 901–905. 12434958 PMC1314441

[ref98] Miller F. G. Colloca L. (2009). The legitimacy of placebo treatments in clinical practice: evidence and ethics. Am. J. Bioeth. 9, 39–47. doi: 10.1080/15265160903316263, PMID: 20013499

[ref99] Miller F. G. Colloca L. (2010). Semiotics and the placebo effect. Perspect. Biol. Med. 53, 509–516. doi: 10.1353/pbm.2010.0004, PMID: 21037405

[ref100] Moore R. Cai N. Skljarevski V. Tölle T. (2014). Duloxetine use in chronic painful conditions–individual patient data responder analysis. Eur. J. Pain 18, 67–75. doi: 10.1002/j.1532-2149.2013.00341.x, PMID: 23733529 PMC4302330

[ref101] Murdoch B. Carr S. Caulfield T. (2016). Selling falsehoods? A cross-sectional study of Canadian naturopathy, homeopathy, chiropractic and acupuncture clinic website claims relating to allergy and asthma. BMJ Open 6:e014028. doi: 10.1136/bmjopen-2016-014028, PMID: 27986744 PMC5168671

[ref102] Narayanaswami P. Gronseth G. Dubinsky R. Penfold-Murray R. Cox J. Bever C.. (2015). The impact of social media on dissemination and implementation of clinical practice guidelines: a longitudinal observational study. J. Med. Internet Res. 17:e193. doi: 10.2196/jmir.4414, PMID: 26272267 PMC4736287

[ref103] National Health Medical Research Council (2015). NHMRC information paper: evidence on the effectiveness of homeopathy for treating health conditions. Canberra, Australia: National Health and Medical Research Council.

[ref104] Nitzan U. Carmeli G. Chalamish Y. Braw Y. Kirsch I. Shefet D.. (2020). Open-label placebo for the treatment of unipolar depression: results from a randomized controlled trial. J. Affect. Disord. 276, 707–710. doi: 10.1016/j.jad.2020.07.077 32871704

[ref105] Oberbaum M. Vithoulkas G. Van Haselen R. (2003). Clinical trials of classical homeopathy: reflections on appropriate research designs. J. Altern. Complement. Med. 9, 105–111. doi: 10.1089/107555303321222982, PMID: 12676039

[ref106] Ostermann T. Burkart J. De Jaegere S. Raak C. Simoens S. (2024). Overview and quality assessment of health economic evaluations for homeopathic therapy: an updated systematic review. Expert Rev. Pharmacoecon. Outcomes Res. 24, 117–142. doi: 10.1080/14737167.2023.2266136, PMID: 37795998

[ref107] Ostermann J. K. Reinhold T. Witt C. M. (2015). Can additional homeopathic treatment save costs? A retrospective cost-analysis based on 44500 insured persons. PLoS One 10:e0134657. doi: 10.1371/journal.pone.0134657, PMID: 26230412 PMC4521756

[ref108] Park C. Pagnini F. Reece A. Phillips D. Langer E. (2016). Blood sugar level follows perceived time rather than actual time in people with type 2 diabetes. Proc. Natl. Acad. Sci. 113, 8168–8170. doi: 10.1073/pnas.1603444113, PMID: 27382161 PMC4961154

[ref109] Petrie K. J. Rief W. (2019). Psychobiological mechanisms of placebo and nocebo effects: pathways to improve treatments and reduce side effects. Annu. Rev. Psychol. 70, 599–625. doi: 10.1146/annurev-psych-010418-102907, PMID: 30110575

[ref110] Posadzki P. Alotaibi A. Ernst E. (2012). Adverse effects of homeopathy: a systematic review of published case reports and case series: the safety of homeopathy. Int. J. Clin. Pract. 66, 1178–1188. doi: 10.1111/ijcp.12026, PMID: 23163497

[ref111] Reid S. (2002). A survey of the use of over-the-counter homeopathic medicines purchased in health stores in Central Manchester. Homeopathy 91, 225–229. doi: 10.1054/homp.2002.0053, PMID: 12422926

[ref112] Relton C. Cooper K. Viksveen P. Fibert P. Thomas K. (2017). Prevalence of homeopathy use by the general population worldwide: a systematic review. Homeopathy 106, 69–78. doi: 10.1016/j.homp.2017.03.002, PMID: 28552176

[ref113] Rief W. Avorn J. Barsky A. J. (2006). Medication-attributed adverse effects in placebo groups: implications for assessment of adverse effects. Arch. Intern. Med. 166, 155–160. doi: 10.1001/archinte.166.2.155 16432082

[ref114] Rief W. Glombiewski J. A. (2012). The hidden effects of blinded, placebo-controlled randomized trials: an experimental investigation. Pain 153, 2473–2477. doi: 10.1016/j.pain.2012.09.007, PMID: 23084328

[ref115] Rief W. Nestoriuc Y. von Lilienfeld-Toal A. Dogan I. Schreiber F. Hofmann S. G.. (2009a). Differences in adverse effect reporting in placebo groups in SSRI and tricyclic antidepressant trials. Drug Saf. 32, 1041–1056. doi: 10.2165/11316580-000000000-00000, PMID: 19810776

[ref116] Rief W. Nestoriuc Y. Weiss S. Welzel E. Barsky A. J. Hofmann S. G. (2009b). Meta-analysis of the placebo response in antidepressant trials. J. Affect. Disord. 118, 1–8. doi: 10.1016/j.jad.2009.01.029 19246102

[ref117] Roji R. Stone P. Ricciardi F. Candy B. (2020). Placebo response in trials of drug treatments for cancer-related fatigue: a systematic review, meta-analysis and meta-regression. BMJ Support. Palliat. Care 10, 385–394. doi: 10.1136/bmjspcare-2019-002163, PMID: 32046962 PMC7691807

[ref118] Rossettini G. Camerone E. M. Carlino E. Benedetti F. Testa M. (2020). Context matters: the psychoneurobiological determinants of placebo, nocebo and context-related effects in physiotherapy. Arch. Physiother. 10:11. doi: 10.1186/s40945-020-00082-y, PMID: 32537245 PMC7288522

[ref119] Rutherford B. R. Tandler J. Brown P. J. Sneed J. R. Roose S. P. (2014). Clinic visits in late-life depression trials: effects on signal detection and therapeutic outcome. Am. J. Geriatr. Psychiatry 22, 1452–1461. doi: 10.1016/j.jagp.2013.09.003, PMID: 24200597 PMC4009389

[ref120] Rutten L. (A. L. B.) (2013). The importance of case histories for accepting and improving homeopathy. Complement. Ther. Med. 21, 565–570. doi: 10.1016/j.ctim.2013.10.001, PMID: 24280462

[ref121] Ruusuvuori J. (2005). Comparing homeopathic and general practice consultations: the case of problem presentation. Commun. Htmlent Glyphamp Asciiamp Med. 2, 123–135. doi: 10.1515/come.2005.2.2.123, PMID: 16808718

[ref122] Schaefer M. Harke R. Denke C. (2016). Open-label placebos improve symptoms in allergic rhinitis: a randomized controlled trial. Psychother. Psychosom. 85, 373–374. doi: 10.1159/000447242, PMID: 27744433

[ref123] Schaefer M. Sahin T. Berstecher B. (2018). Why do open-label placebos work? A randomized controlled trial of an open-label placebo induction with and without extended information about the placebo effect in allergic rhinitis. PLoS One 13, e0192758–e0192714. doi: 10.1371/journal.pone.0192758, PMID: 29513699 PMC5841659

[ref124] Schafer S. M. Colloca L. Wager T. D. (2015). Conditioned placebo analgesia persists when subjects know they are receiving a placebo. J. Pain 16, 412–420. doi: 10.1016/j.jpain.2014.12.008, PMID: 25617812 PMC4424173

[ref125] Schedlowski M. Enck P. Rief W. Bingel U. (2015). Neuro-bio-behavioral mechanisms of placebo and nocebo responses: implications for clinical trials and clinical practice. Pharmacol. Rev. 67, 697–730. doi: 10.1124/pr.114.009423, PMID: 26126649

[ref126] Scherer L. D. McPhetres J. Pennycook G. Kempe A. Allen L. A. Knoepke C. E.. (2021). Who is susceptible to online health misinformation? A test of four psychosocial hypotheses. Health Psychol. 40, 274–284. doi: 10.1037/hea0000978, PMID: 33646806

[ref127] Shapiro L. A. (2011). Embodied cognition. London, New York: Routledge.

[ref128] Shapiro L. A. (2014). The Routledge handbook of embodied cognition. London: Routledge.

[ref129] Sigurdson M. K. Sainani K. L. Ioannidis J. P. A. (2023). Homeopathy can offer empirical insights on treatment effects in a null field. J. Clin. Epidemiol. 155, 64–72. doi: 10.1016/j.jclinepi.2023.01.010, PMID: 36736709

[ref130] Sondermann W. Reinboldt-Jockenhöfer F. Dissemond J. Pfaar O. Bingel U. Schedlowski M. (2021). Effects of patients’ expectation in dermatology: evidence from experimental and clinical placebo studies and implications for dermatologic practice and research. Dermatology 237, 857–871. doi: 10.1159/000513445, PMID: 33498052

[ref131] Swayne J. (2000). International dictionary of homeopathy. Edinb. Lond: Churchill Livingstone, 222.

[ref132] Teixeira M. Z. (2023). Homeopathic Materia Medica of modern drugs. 2nd Edn. São Paulo, Brazil: Portal de Livros Abertos da USP.

[ref133] Thompson E. Dahr J. Susan M. Barron S. (2007). Setting standards in homeopathic practice—a pre-audit exploring motivation and expectation for patients attending the Bristol homeopathic hospital. Homeopathy 96, 243–246. doi: 10.1016/j.homp.2007.08.012, PMID: 17954381

[ref134] Thompson T. D. Weiss M. (2006). Homeopathy – what are the active ingredients? An exploratory study using the UK Medical Research Council’s framework for the evaluation of complex interventions. BMC Complement. Altern. Med. 6:37. doi: 10.1186/1472-6882-6-37, PMID: 17101037 PMC1676018

[ref135] Ulrich R. S. (1984). View through a window may influence recovery from surgery. Science 224, 420–421. doi: 10.1126/science.6143402, PMID: 6143402

[ref136] Uluocak A. (2023). Menopausal period and homeopathy: a review. J. Controv. Obstet. Gynecol. Pediatr. 1, 46–50. doi: 10.51271/JCOGP-0011

[ref137] Vase L. Vollert J. Finnerup N. B. Miao X. Atkinson G. Marshall S.. (2015). Predictors of the placebo analgesia response in randomized controlled trials of chronic pain: a meta-analysis of the individual data from nine industrially sponsored trials. Pain 156, 1795–1802. doi: 10.1097/j.pain.0000000000000217 25955965

[ref138] von Wernsdorff M. Loef M. Tuschen-Caffier B. Schmidt S. (2021). Effects of open-label placebos in clinical trials: a systematic review and meta-analysis. Sci. Rep. 11:3855. doi: 10.1038/s41598-021-83148-6, PMID: 33594150 PMC7887232

[ref139] Waber R. L. (2008). Commercial features of placebo and therapeutic efficacy. JAMA 299, 1016–1017. doi: 10.1001/jama.299.9.1016, PMID: 18319411

[ref140] Waisse S. (2021). Severe acute Thromboinflammation: case report of individualized homeopathic treatment. Homeopathy 110, 132–136. doi: 10.1055/s-0040-1721064, PMID: 33618380

[ref141] Walach H. Jonas W. B. Ives J. Wijk R. V. Weingärtner O. (2005). Research on homeopathy: state of the art. J. Altern. Complement. Med. 11, 813–829. doi: 10.1089/acm.2005.11.813, PMID: 16296915

[ref142] Weimer K. Colloca L. Enck P. (2015). Placebo effects in psychiatry: mediators and moderators. Lancet Psychiatry 2, 246–257. doi: 10.1016/S2215-0366(14)00092-3, PMID: 25815249 PMC4370177

[ref143] Weinberger J. M. Houman J. Caron A. T. Patel D. N. Baskin A. S. Ackerman A. L.. (2018). Female sexual dysfunction and the placebo effect: a meta-analysis. Obstet. Gynecol. 132, 453–458. doi: 10.1097/AOG.0000000000002733 29995725

[ref144] Whitmont R. D. (2019). “Homeopathy for gastrointestinal disorders” in Integrative gastroenterology. eds. Mullin G. E. Singh M. Parian A. Clarke J. Mullin G. E. Singh M.. (New York, NY: Oxford University Press).

[ref145] Wilhelm M. Euteneuer F. (2021). Does health literacy make a difference? Comparing the effect of conventional medicine versus homeopathic prescribing on treatment credibility and expectancy. Front. Psychol. 12:581255. doi: 10.3389/fpsyg.2021.581255, PMID: 34140910 PMC8204743

[ref146] Wilhelm M. Rief W. Doering B. K. (2018). Decreasing the burden of side effects through positive message framing: an experimental proof-of-concept study. Int. J. Behav. Med. 25, 381–389. doi: 10.1007/s12529-018-9726-z, PMID: 29785686

[ref147] Wilhelm M. Winkler A. Rief W. Doering B. K. (2016). Effect of placebo groups on blood pressure in hypertension: a meta-analysis of beta-blocker trials. J. Am. Soc. Hypertens. 10, 917–929. doi: 10.1016/j.jash.2016.10.009, PMID: 27865824

[ref148] Winkler A. Rief W. (2015). Effect of placebo conditions on polysomnographic parameters in primary insomnia: a Meta-analysis. Sleep 38, 925–931. doi: 10.5665/sleep.4742, PMID: 25515108 PMC4434559

[ref01] Winkler A. Hahn A. Hermann C. (2023). The impact of pharmaceutical form and simulated side effects in an open-label-placebo RCT for improving psychological distress in highly stressed students. Scientific Reports 13:6367. doi: 10.5281/zenodo.7845278, PMID: 37076557 PMC10113726

[ref149] Yetman H. E. Cox N. Adler S. R. Hall K. T. Stone V. E. (2021). What do placebo and nocebo effects have to do with health equity? The hidden toll of nocebo effects on racial and ethnic minority patients in clinical care. Front. Psychol. 12:788230. doi: 10.3389/fpsyg.2021.788230, PMID: 35002881 PMC8733207

[ref150] Zech N. Scharl L. Seemann M. Pfeifer M. Hansen E. (2022). Nocebo effects of clinical communication and placebo effects of positive suggestions on respiratory muscle strength. Front. Psychol. 13:825839. doi: 10.3389/fpsyg.2022.825839 35360592 PMC8962828

[ref151] Zhou E. S. Hall K. T. Michaud A. L. Blackmon J. E. Partridge A. H. Recklitis C. J. (2018). Open-label placebo reduces fatigue in cancer survivors: A randomized trial. Support Care Cancer 27, 2179–2187. doi: 10.1007/s00520-018-4477-6 30298411

